# Editorial: Host-pathogen interactions: cellular damage, death, and adaptation in microbial infections

**DOI:** 10.3389/fcimb.2026.1852001

**Published:** 2026-05-29

**Authors:** Chaitenya Verma, Nawaid Hussain Khan, Anuradha Tyagi, Kavita Arora, Vinay Kumar

**Affiliations:** 1 Department of Biotechnology, School of Bio-Science and Technology, Sharda University Greater Noida, Uttar Pradesh, India; 2 Faculty of Medicine, Ala-Too International University, Bishkek, Kyrgyzstan; 3 Department of cBRN, Institute of Nuclear Medicine & Allied Sciences, Delhi, India; 4 Advanced Instrumentation Research Facility, Jawaharlal Nehru University, Delhi, India; 5 College of Medicine, Pennsylvania State University Hershey Medical Center, Hershey, PA, United States

**Keywords:** adaptation, cell death, cellular damage, host-pathogen interactions, microbial infections

## Introduction

In recent years, scientists have worked on how microbial pathogens are able to manipulate host defence machinery, including immunological signalling pathways, to attain persistence and elude elimination. This Research Topic’s articles from various studies focus on interwoven themes of cellular stress, immunological cellular modulation, and diagnostic innovation, advocating for a paradigm shift toward targeted therapies.

## Strategies for emerging pathogens

Pathogens efficiently practice a variety of methods for their survival and to evade cellular immune mechanisms. Gram-negative bacterial pathogens such as Salmonella enterica and Vibrio parahaemolyticus specifically target mitochondria, leading to cellular stress that disrupts oxidative phosphorylation and the balance of reactive oxygen species (ROS) (Liu et al., 2020), ultimately resulting in dysfunctional mitophagy (Liu et al., 2024). This “missing link” connects acute infection to post-infectious metabolic diseases, such as insulin resistance and chronic inflammation, even after therapeutic intervention. This suboptimal immune restoration is due to unresolved cellular stress that causes long-lasting immunometabolic alteration in epithelial and adipose tissues (Plaza et al.). Similarly, the World Health Organization (WHO) identified *Klebsiella pneumoniae* as one of the top 10 serious global public health threats in the antimicrobial resistance (AMR) gram-negative category that contributes to 39% of susceptible ICU patients.

Viral infections exaggerate this impediment and endorse immunometabolic alteration (Verma et al., 2019). Additionally, atmospheric particulate matter (PM) exposure, especially PM-2.5 to PM-10 particles, increases respiratory morbidity and mortality, which is directly associated with the downregulation of epithelial apoptosis and cytokines like IFN-γ (Hamanaka, 2025). Even during the COVID-19 pandemic, epidemiological studies found that pre-exposure to PM may worsen SARS-CoV-2 infection aetiology and treatment outcome, although the exact cellular mechanism remains unclear (Travaglio et al., 2021). A few studies found PM’s effects on SARC-CoV-2 infection kinetics in Calu-3 human epithelial cells when exposed to PM for 72 hr; cells were infected with the wild type Wuhan strain or the more virulent Delta strain without influencing ACE2 entry (Kongsomros et al.).

Toscana virus strains shows a lot of differences. The long-lasting INMI variant stops the first IFN responses in brain organoids, which makes replication last 21 days by delaying NSs protein expression. This is different from the self-limiting SI-1812. In Drosophila models of porcine rotavirus, JAK-STAT activation elevates reactive oxygen species and antimicrobial peptides, equilibrating gut epithelial apoptosis with hyperplasia amid microbiome modifications and RNA interference evasion (Wang et al.).

## Cellular mortality and immune reconfiguration

Programmed cell death functions as a dual-edged weapon in the context of infections. Transcriptome profiling of osteomyelitis by GSVA exposes multiple programmed cell death pathways like apoptosis, autophagy, cuproptosis, and entosis, which is accompanied by immune infiltration, resulting in hub genes (SORT1, KIF1B, TMEM106B, NPC1, ATP6V0B) confirmed by qPCR for early diagnostic purposes (Feng et al.). These formerly neglected non-classical deaths now highlight new treatment targets in osteomyelitis.

Immune profiling improves the disclosure; leptospirosis increases CCL5, CXCL5, and CXCL9 levels, which are in line with the changes in CXCL11, CCL3, and CCL2. This makes CCL5 a diagnostic biomarker for this 15% fatal zoonosis that affects one million individuals annually (Mariano et al.). Nontuberculous mycobacteria (NTM), now including over 200 species, have evolved from diagnostic obscurity to prominence via molecular techniques like whole-genome sequencing, requiring customized treatment regimens post-sputum conversion (Sharma et al.). Polymorphisms of the vitamin D receptor (FokI and TaqI) amplify the risks of multidrug-resistant tuberculosis in high-burden regions such as India, interlinking genetics, nutrition, and resistance (Rathored et al.).

## Diagnostic and therapeutic perspectives

These studies focus on precision medicine. Historical insights from NTM-taxonomy challenges and cultural biases-are now integrated with line-probe assays for patient-centred care. Biomarkers from VDR mutants and chemokines allow for faster diagnosis of MDR-TB than traditional DST, which is slow and ineffective. PCD hubs, on the other hand, help separate different types of osteomyelitis. Fly intestines replicate porcine rotavirus defences, confirming Drosophila as an effective model for antiviral immunity. However, significant gaps persist, and findings from *in vitro* organoid models needed *in vivo* validation; the rise of resistance epidemics (*K. pneumonia* and MDR-TB) requires comprehensive solutions.

## A request for engagement

This editorial highlights a biological conflict where pathogens use host machinery like mitochondrial centres, programmed cell death pathways, and mitigated cytokine and chemokine profiles lead to their supremacy. Mitochondria-focused therapies may diminish Salmonella’s metabolic impact, and moderating particulate matter, especially PM10 impact, can improve post-COVID environments. Under other conditions, targeting nonstructural proteins (NSs) among neuroviruses, particularly TOSV strains, can be used for the development of vaccines by activating endogenous retinoic acid-inducible gene-I (RIG-1), and modulation in chemokine panels enhances the triage of leptospirosis.

Integration of interdisciplinary essentials like genomics, transcriptomics, and various novel disease models are needed to elucidate pathogen resistance mechanisms. Policymakers should prioritise quality lifestyles, VDR screenings in endemic regions, and NTM surveillance. As zoonoses increase, such as leptospirosis and rotaviruses, these discoveries equip us to combat the perseverance of pathogens in addition to establishing robust defences based on cellular knowledge.

In conclusion, research on these Research Topic has led to a better understanding of how pathogens invade and identifying anticipatory measures that mitigate chronic consequences.
